# Navigating the role of protein lactylation in prostate cancer and its implications for immunotherapy

**DOI:** 10.7150/jca.114137

**Published:** 2025-06-12

**Authors:** Dongzhang Li, Kaifeng Wang, Guantao Lou, Wangjian Li, Quan Ma, Yu'e Liu, Yongliang Chen

**Affiliations:** 1 Dept of Urology, Shaoxing Central Hospital, Shaoxing, Zhejiang, 312030, China.; 2 Dept of Urology, The Central Affiliated Hospital of Shaoxing University, Shaoxing, Zhejiang, 312030, China.; 3 Boston Children's Hospital, Dana Farber Cancer Institute, Harvard Medical School, Boston, Massachusetts 02115, USA.; 4 Department of Neurosurgery, Shanghai East Hospital, School of Medicine, Tongji University, Shanghai, 200120, China.

**Keywords:** lactylation, prostate cancer, immunotherapy, immune checkpoints, tumor microenvironment

## Abstract

Prostate cancer is an aggressive malignancy with high prevalence and significant mortality, characterized by its remarkable metabolic adaptability and immune complexity. Emerging evidence has highlighted the critical role of post-translational modifications (PTMs) in cancer biology, with protein lactylation gaining attention as a novel PTM with profound implications. Lactylation, derived from lactate, links the altered metabolic processes of tumor cells to diverse cellular functions, including epigenetic regulation and protein dynamics. It significantly influences tumor progression, immune evasion, and therapeutic resistance by modulating key immune cells within the tumor microenvironment. The immunosuppressive conditions created by lactate and lactylation favor tumor survival in prostate cancer. Thus, targeting lactylation offers innovative strategies for treating prostate cancer. By leveraging lactylation modulation, particularly in combination with immune checkpoint inhibitors, there is potential to enhance anti-tumor immune responses and improve treatment outcomes. This review explores the intersection of metabolic alterations and immune modulation, underscoring lactylation as a promising therapeutic avenue in prostate cancer.

## 1. Introduction

Prostate cancer is one of the most prevalent malignancies worldwide 1, 2. Despite advancements in diagnosis and treatment, prostate cancer remains a leading cause of cancer-related mortality. It is characterized by its heterogeneity and immunosuppressive nature, ranging from localized forms to aggressive and metastatic variants 3, 4. The main factors contributing to its progression are intricate interplays of genetic, metabolic, and immunological elements. These interconnections not only drive tumor growth and metastasis but also contribute to the development of therapeutic resistance, posing significant challenges. One of the hallmarks of prostate cancer is its ability to reprogram cellular metabolism to support rapid proliferation and survival in hostile microenvironments 5. Prostate cancer cells frequently exhibit a metabolic shift, transitioning from oxidative phosphorylation to increased glycolysis and lipid metabolism, even under aerobic conditions 6. This phenomenon, often termed the "Warburg effect," generates metabolic byproducts such as lactate, which were once considered mere waste products but are now recognized as active participants in cancer biology 7. The accumulation of lactate in the tumor microenvironment (TME) influences cellular signaling, immune regulation, and epigenetic reprogramming profoundly 8, 9. Thus, lactate is regarded as a key metabolite bridging metabolic and immunological landscapes in prostate cancer 10.

**Figure 1:**
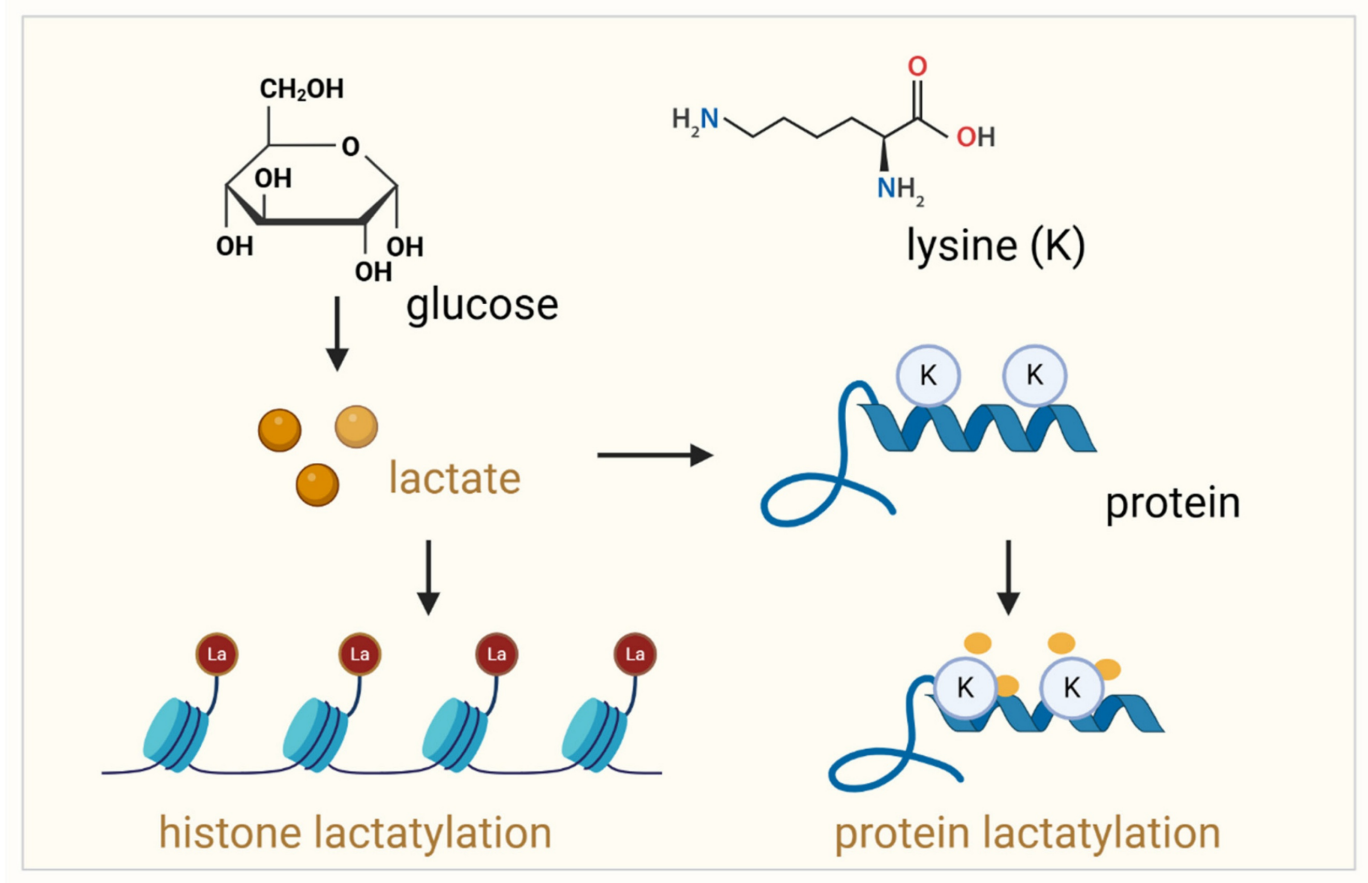
** Lactylation of histone proteins and non-histone proteins.** This schematic illustrates the process of lactylation, a post-translational modification where lactate is covalently added to lysine (Lys) residues in proteins. On the left, lactylation of histone proteins is shown, where lactate, produced from glucose metabolism, is transferred to lysine residues in the histone tails, influencing chromatin structure and gene expression. The right panel depicts the lactylation of non-histone proteins, where lactate binds to lysine residues of non-histone proteins, altering their function, stability, and interactions. In both cases, lactate modification can significantly impact protein function and cellular processes such as gene regulation, immune response, and tumor progression. The diagram emphasizes the role of lactate metabolism in regulating protein modifications and their implications in various biological contexts.

Protein lactylation is a novel discovered post-translational modification (PTM), which involves the addition of lactyl groups to lysine residues on proteins. It is a process mediated by lactate-derived metabolites 11-13 (Figure 1). It usually occurs on lysine residues of amino acids. Lysine lactylation has three types: L-lysine lactylation (KL-la), D-lysine lactylation (KD-la), and N-ε-carboxyethyl lysine (Kce). KL-la, the predominant form, involves the addition of an L-lactyl group and regulates processes like gene expression under glycolytic conditions 14. KD-la, less common, adds a D-lactyl group through non-enzymatic reactions and is implicated in cancer and neurological disorders. Kce, structurally distinct, arises from the addition of a carboxyethyl group, often linked to aging and chronic diseases as an advanced glycation end product 15. Advanced mass spectrometry techniques enable differentiation and deeper exploration of their specific biological roles. Lactylation modifications use lactyl-CoA as the L-lactate donor and primarily occur at nucleophilic sites, such as amino groups (-NH2), through interactions with corresponding functional groups to precisely target specific proteins 16. Similar to other PTMs, lactylation has its own “writer,” “reader,” and “eraser” proteins, which regulate its addition, recognition, and removal, respectively 15. Unlike classical PTMs such as phosphorylation or acetylation, lactylation establishes a direct biochemical link between metabolic reprogramming and protein-level regulation 14. This emerging modification has profound implications for cancer biology, particularly in prostate cancer, where metabolic adaptation and immune evasion are critical to cancer progression 17. In prostate cancer, lactylation has been implicated in several oncogenic processes, including tumor proliferation, angiogenesis, and immune modulation. It influences gene expression through chromatin remodeling, impacts signaling pathways essential for cell survival, and shapes the immune landscape by modulating the activity of immune cells 18, 19. For instance, lactylation-mediated changes in macrophages within the tumor microenvironment polarize them towards a tumor-promoting M2 phenotype, facilitating immune evasion and therapeutic resistance 11, 20. Therefore, lactylation is a mediator of crosstalk between cancer metabolism and immune responses.

In addition, protein lactylation in prostate cancer is closely related to immunotherapy. While immune checkpoint inhibitors (ICIs) have revolutionized the treatment landscape for several cancers, their efficacy in prostate cancer has been modest 21. The immunologically “cold” nature of prostate tumors, characterized by low immune cell infiltration and an immunosuppressive microenvironment, limits the effectiveness of ICIs 21, 22. In metastatic castration-resistant prostate cancer (mCRPC), the tumor microenvironment influences immune checkpoint therapy (ICT) efficacy. In subcutaneous tumors, ICT increases Th1 cells and improves survival, while in bone metastases, it fails due to Th17 polarization driven by TGF-β from osteoclast-mediated bone resorption. Blocking TGF-β restores Th1 responses, enhances CD8+ T cell expansion, and improves ICT efficacy, offering a strategy to overcome resistance in bone-metastatic mCRPC^23^. The high levels of lactate and lactylation contribute to the immunosuppressive TME of prostate cancer. Targeting lactylation modulates immune responses and reshapes the TME, enhancing immunogenicity, improving immune cell recruitment and activation, and potentiating the effects of ICIs 11. Beyond immunotherapy, lactylation holds potential as a biomarker for prostate cancer progression and therapeutic response. Its metabolic origins make it a dynamic indicator of tumor activity, reflective of changes in the TME. Additionally, targeting lactylation through inhibitors of lactate production or enzymes involved in lactylation could open new therapeutic windows.

This review aims to provide an updated exploration of protein lactylation in prostate cancer, integrating insights from metabolic, epigenetic, and immunological perspectives. We will delve into the mechanisms underlying lactylation, its functional impacts on tumor and immune cells, and its potential as a therapeutic target. By bridging the metabolic and immunological domains, this discussion seeks to offer new perspectives on leveraging lactylation to advance prostate cancer treatment, particularly in the combination of immunotherapy.

## 2. Protein Lactylation

### 2.1. Protein lactylation regulation and functions

Protein lactylation is regulated by a series of proteins. Lactylation “writers” are enzymes that catalyze the lactylation process, facilitating the interaction between lactate and target proteins, leading to the incorporation of lactyl groups. Recent studies have identified several acetyltransferases, including p300 20, KAT2A 24, and KAT5 25 as key enzymes involved in histone lactylation. Histone acetyltransferases (HATs) and sirtuins are the writers and erasers for histone lactylation 26. SIRT1/SIRT3 have been proven to be robust lysine delactylases and SIRT1-mediated delactylation regulates glycolysis 27. Sirtuin 3 also mediates the delactylation of the cell cycle protein E2 (CCNE2), and the absence of lactylation on CCNE2 suppresses liver cancer growth 26. SIRT1-mediated delactylation of PTBP1 promotes glioma stem cell maintenance by enhancing PFKB4-driven glycolysis 28.

Lactylation was first identified as a histone modification that promotes gene expression in response to increased lactate levels, a hallmark of the Warburg effect in cancer 20. By adding a lactyl group derived from lactate to lysine residues on proteins, lactylation alters their structure, stability and function. For example, lactylation of β-catenin protein enhances its stability and facilitates its cellular entry via MCT1 in prostate cancer cells 29. Additionally, increasing the lactylation level of HIF-1α can stabilize HIF-1α under normoxic conditions 29. Beyond these, other signaling proteins and transcription factors have also been implicated. The lactylation of SOX9 promotes its activity, enhancing cell stemness, migration, and invasion. In tumor-bearing mice, overexpression of SOX9 accelerates tumor growth, while inhibition of glycolysis reverses this effect and suppresses tumor progression 30. The lactylation of TFEB at lysine 91 stabilizes the protein by preventing its interaction with the E3 ubiquitin ligase WWP2, inhibiting its ubiquitination and subsequent degradation. This modification enhances TFEB's role in promoting autophagy, with implications for cancer, where increased TFEB lactylation may contribute to elevated autophagic activity in tumor cells 31. Thus, lactylation of signaling proteins and transcription factors alters their localization or interaction with other molecules, impacting pathways that regulate cell survival, proliferation, and apoptosis 32, 33. Histone lactylation activates oncogenic pathways or suppresses tumor-suppressor genes, facilitating tumor progression. Beyond histones, non-histone protein lactylation has been implicated in diverse cellular processes, including metabolic regulation, immune modulation, and cancer development 34. P53 lactylation at lysine 120 and lysine 139 mediated by AARS1 promotes tumorigenesis and progression 35. Furthermore, proteins involved in metabolic pathways, such as glycolysis and the tricarboxylic acid (TCA) cycle, undergo lactylation, influencing their enzymatic activity and metabolic flux 36, 37. This modulation ensures that metabolic outputs align with the energetic and biosynthetic demands of rapidly proliferating cancer cells.

Protein lactylation plays a pivotal role in shaping TME and regulating immune response 38. Lactylation of transcription factors or signaling mediators in immune cells reprogram their activity, promoting either pro-inflammatory or immunosuppressive phenotypes 35. Lactylation also influences the activity of cytotoxic T cells and natural killer (NK) cells, two critical components of the anti-tumor immune response. H3K18la and H3K9la in CD8^+^ T cell subsets are linked to their specific metabolic profiles. By targeting metabolic and epigenetic pathways, these modifications influence CD8^+^ T cell effector functions, including antitumor immunity 39, 40. By modulating the expression of immune checkpoints or altering metabolic pathways within these cells, lactylation diminishes their cytolytic activity, contributing to immune evasion. H3K18 lactylation activated POM121, which enhanced MYC nuclear translocation and direct binding to the CD274 to potentiate PD-L1 expression and finally potentiates immune escape of lung cancer 41.

### 2.2. Metabolic reprogramming and lactylation in prostate cancer

**Figure 2:**
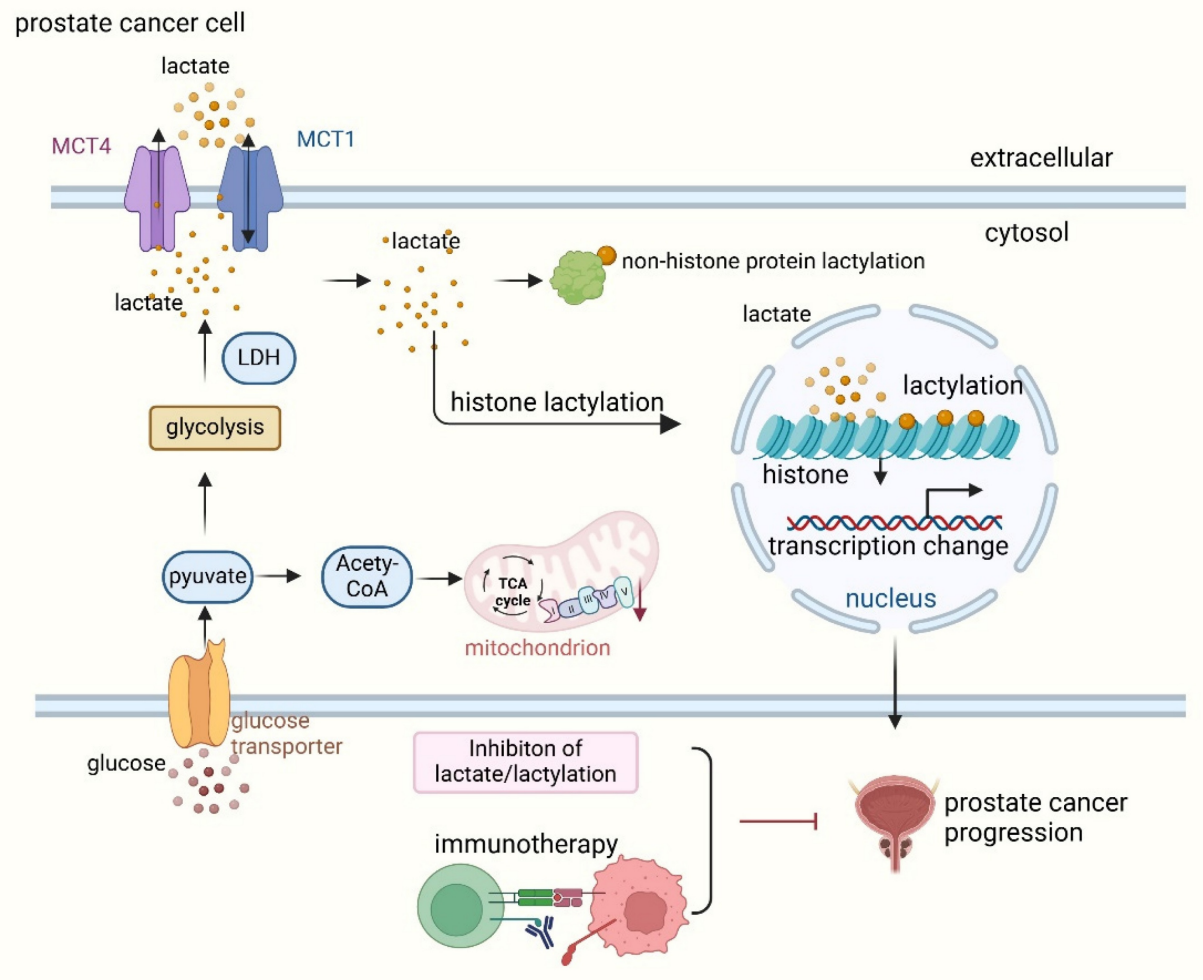
** Lactate and lactylation promote prostate cancer progression.** Lactate is transported into prostate cancer cells via monocarboxylate transporter 1 (MCT1) and exported to the extracellular space by MCT4. To meet the high energy demands during progression, prostate cancer cells primarily rely on glycolysis, which generates pyruvate. Lactate dehydrogenase (LDH) catalyzes the conversion of pyruvate to lactate. Elevated lactate levels lead to both histone and non-histone lactylation, which subsequently promotes prostate cancer progression. Reducing lactate levels and inhibiting lactylation, especially in combination with immunotherapy, synergistically suppress prostate cancer progression.

Prostate cancer cells exhibit profound metabolic reprogramming, characterized by increased glycolysis and lactate production. Lactate is secreted by cancer-associated fibroblasts (CAF) and taken up by prostate cancer cells to support mitochondrial metabolism. CAF-secreted lactate increases the expression of lipid metabolism genes in cancer cells, enhances lipid accumulation in lipid droplets (LDs) and provides acetyl groups for histone acetylation, creating a feedback loop between metabolites and epigenetic modifications 7. These metabolic shifts create a microenvironment rich in lactate, which not only fuels tumor progression but also drives protein lactylation (Figure 2).

#### 2.2.1. Lactate synthesis in prostate cancer

Lactate is synthesized by lactate dehydrogenases (LDHs), which consist of two main subunits: LDHA and LDHB. These subunits form homotetrameric isoforms (LDH1 and LDH5) or heterotetrameric isoforms (LDH2, LDH3, and LDH4), all of which catalyze the conversion between pyruvate and lactate. LDHA primarily converts pyruvate to lactate, supporting glycolysis, while LDHB facilitates the conversion of lactate to pyruvate 42. A third isoform, LDHC, is expressed in human testes and plays a role in male fertility 43. Elevated LDHA levels have been linked to poor prognosis in prostate cancer, with high LDHA and low LDHB levels associated with shorter survival and quicker recurrence 8. FGFR1 regulates their expression by stabilizing LDHA protein through phosphorylation while repressing LDHB transcription 44. High serum LDHA is a poor prognostic marker in metastatic prostate cancer. To manage lactate levels, cancer cells use monocarboxylate transporters (MCTs) to export lactate into the TME, with MCT-1 involved in lactate import and MCT-4 in lactate efflux. MCT-4 expression in PCa cells is linked to higher Gleason scores, advanced stages, and biochemical recurrence, further indicating its role in tumor progression.

#### 2.2.2. Lactate shuttling between glycolytic CAF and oxidative prostate cancer cells enhances tumor progression

Castration-resistant prostate cancer cells increase aerobic glycolysis and lactate secretion to promote metastasis and an immunosuppressive TME 45. Lactate is secreted by CAFs and taken up by prostate cancer cells to support mitochondrial metabolism. CAF-secreted lactate increases the expression of lipid metabolism genes in cancer cells 7. In hypoxic, poorly vascularized areas, glycolytic cancer cells and CAFs convert glucose to lactate, which is then exported via MCT-4 to more oxygenated areas 46. Oxidative prostate cancer cells take up lactate through MCT-1, converting it to pyruvate for energy production through the TCA cycle. This lactate shuttling between glycolytic and oxidative cells sustains tumor growth in nutrient-deprived environments. Oxidative cancer cells instruct CAFs to enhance glycolysis, a phenomenon known as the "reverse Warburg effect." Inhibition of MCT-1 reduces prostate cancer survival under glucose restriction, confirming the importance of lactate shuttling. The increased MCT-4 in CAFs and MCT-1 in prostate cells is associated with cancer progression and recurrence. In addition, lactate uptake increases the NAD^+^/NADH ratio in prostate cancer cells, promoting mitochondrial changes and aggressiveness. Additionally, lactate boosts de novo fatty acid synthesis in CAFs, leading to lipid accumulation and histone acetylation that drive prostate cancer progression 7. Inhibiting the CAF-induced metabolic-epigenetic loop reduces tumor growth and metastasis.

#### 2.2.3. Lactylation promotes prostate cancer progress by orchestrating various gene functions

The increased glycolysis and lactate production in prostate cancer create a microenvironment rich in lactate, which not only fuels tumor progression but also drives protein lactylation. Lactylation-related genes (LRGs) play a significant role in prostate cancer progression by influencing the TME and therapeutic response. High-risk patients, identified using an LRG-based prognostic model, exhibit increased regulatory T cells, M2 macrophages, higher tumor mutation burden, drug resistance and worse prognosis. Key LRGs are notably overexpressed in castration-resistant prostate cells, highlighting their potential as biomarkers for predicting disease-free survival (DFS) and guiding treatment strategies 47. Key metabolic enzymes and transcriptional regulators, such as HIF-1α, may be influenced by lactylation, enhancing their oncogenic potential. Hypoxia-inducible factor 1-alpha (HIF-1α) is a key player in the metabolic adaptation of prostate cancer cells to hypoxic conditions. HIF-1α drives the expression of genes involved in glycolysis, angiogenesis, and survival under low oxygen tension. Lactylation of HIF-1α enhances KIAA1199 transcription further promoting hypoxia-associated oncogenic pathways in prostate cancer 18. Inhibition of HIF-1α suppressed prostate cancer, Evodiamine impairs HIF-1α histone lactylation to inhibit Sema3A-mediated angiogenesis and PD-L1 by inducing prostate cancer cell ferroptosis 19. Similarly, p53 lactylation by NF-κB/STAT3/SLC4A4 axis leads to the development of enzalutamide resistance and progression of prostate cancer 48. Additionally, lactylation of tumor suppressors or DNA repair proteins could contribute to genetic instability and treatment resistance.

### 2.3. Lactylation modulates immune responses in the tumor microenvironment

Lactate accumulation, a hallmark of the altered metabolism in tumors, plays a crucial role in shaping the immune landscape within TME. Elevated levels of lactate result in lactylation which has profound effects on immune cells and their function. In prostate cancer, lactate-induced lactylation significantly influences various immune cell populations, skewing their activity toward a pro-tumorigenic, immunosuppressive phenotype.

#### 2.3.1. Lactate and lactylation regulate macrophage function in prostate cancer

Macrophages are key cells of the innate immune system, responsible for phagocytosis, antigen presentation, and regulation of immune responses, playing critical roles in tissue homeostasis and disease 49, 50. In prostate cancer, tumor-associated macrophages (TAMs) promote tumor progression, angiogenesis, and immune evasion by secreting pro-inflammatory and immunosuppressive factors, thereby exacerbating disease development 51, 52. For example, the YY1 complex in M2 macrophages promotes prostate cancer progression by upregulating IL-6^53^. Lactate-rich conditions in prostate cancer favor the polarization of TAMs towards an M2-like phenotype, characterized by immunosuppressive and pro-tumor properties. This polarization is driven in part by lactylation of key transcriptional regulators in macrophages, which reprogram their function to support tumor progression 11, 20. Blocking lactate production in tumor cells suppresses aggressive PTEN/p53-deficient prostate cancer in mice by enhancing macrophage phagocytosis 54. TAMs also play a critical role in promoting ferroptosis resistance in prostate cancer through their interactions with tumor cells. Disrupting this TAM-mediated crosstalk represents a potential strategy to inhibit tumor progression 55. LXA4 promotes prostate cancer progression by driving M2 macrophage polarization through the suppression of METTL3^56^. In addition, in the prostate cancer TME, CCL2 plays a key role in macrophage polarization by binding to the CCR2 receptor and activating PI3K/Akt signaling 57. CCL2 enhances LPS-induced IL-10 production, while its inhibition promotes M1 polarization and reduces M2 markers 58.

#### 2.3.2. Lactate and lactylation regulate T cell-mediated immune response in prostate cancer

T cells play a crucial role in the immune response to prostate cancer, with CD8^+^ T cells being essential for anti-tumor immunity. In prostate cancer, immune evasion mechanisms, such as T cell exhaustion and a suppressive TME, block effective T cell-mediated tumor elimination, driving cancer progression 59. In T cells, lactate triggers a "stop migration" signal, trapping them at inflammatory sites. This process involves lactate transporters SLC5A12 and SLC16A1 (MCT1), expressed by CD4^+^ and CD8^+^ T cells, respectively 60. Lactate suppresses glycolysis in CD4^+^ T cells by reducing glycolytic enzyme expression and glucose flux, impairing their ability to exit inflamed tissues 61. Lactate also modulates CD4^+^ T cell polarization and induces an immunosuppressive environment, which sustains prostate carcinoma progression via TLR8/miR21 axis 62. Moreover, CAF-derived lactate modulates immune responses, reducing Th1 cells and promoting Tregs, leading to a more invasive phenotype 62. In addition, bone metastasis is common in prostate cancer. Metastatic prostate cancer is characterized by multifaceted immune distortion and exhausted T cells 63.

**Figure 3:**
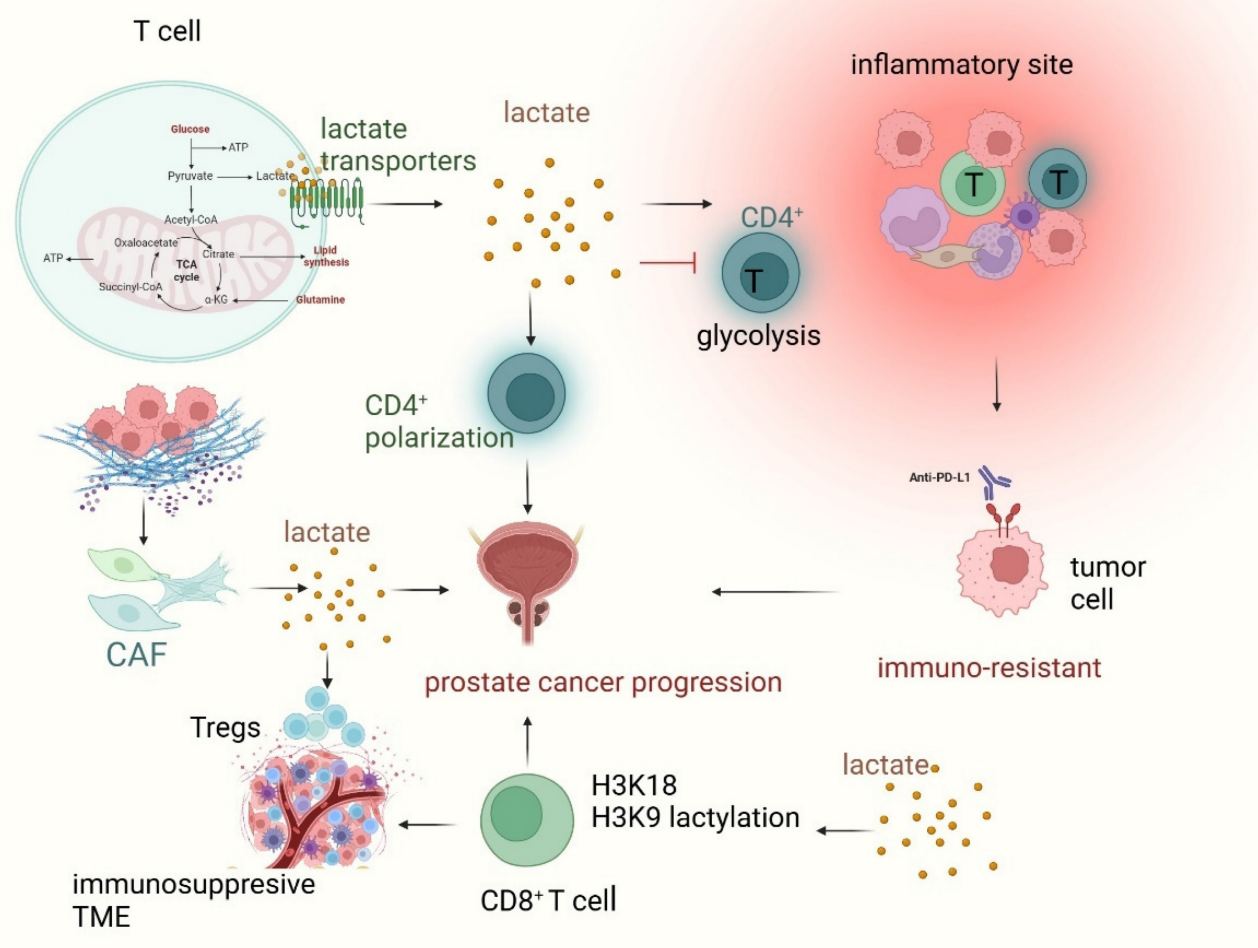
Lactate and lactylation regulate T cell-mediated immune response in prostate cancer. Lactate, a key metabolite in the tumor microenvironment (TME), plays a crucial role in shaping T cell function and immune evasion in prostate cancer. Within T cells, lactate is transported via SLC5A12 (in CD4⁺ T cells) and SLC16A1 (MCT1) (in CD8⁺ T cells), affecting their metabolic activity and immune responses. Increased lactate accumulation inhibits glycolysis in CD4⁺ T cells, impairing their ability to exit inflammatory sites and thereby contributing to immune suppression. Meanwhile, cancer-associated fibroblast (CAF)-derived lactate promotes regulatory T cell (Treg) expansion, creating an immunosuppressive TME that sustains prostate cancer progression. Lactylation, a post-translational modification influenced by lactate metabolism, occurs on histones H3K18 and H3K9 in CD8⁺ T cells, altering gene transcription and leading to T cell exhaustion. This process weakens cytotoxic T cell function, enhancing tumor immune escape and contributing to immune resistance against checkpoint inhibitors like anti-PD-L1 therapy. Overall, lactate and lactylation-mediated metabolic reprogramming promote prostate cancer progression, highlighting potential therapeutic targets for overcoming immunosuppression and enhancing T cell-mediated tumor clearance.

Similarly, lactylation compromises T cell function, contributing to immune evasion. T-cell exhaustion, a state in which T cells lose their ability to effectively recognize and eliminate tumor cells, can be exacerbated by lactylation (Figure 3). Lactylation is observed in both CD8^+^ cytotoxic T cells and CD4^+^ helper T cells, which become less responsive to tumor antigens due to lactate-driven alterations in their signaling pathways and gene expression profiles 64. Lactylation of histones H3K18 and H3K9 in CD8^+^ T cells plays a key role in regulating T cell function by initiating the transcription of genes that control their activity. Distinct patterns of these lactylations are observed in different CD8^+^ T cell subsets, reflecting their specific metabolic profiles. Modulating H3K18la and H3K9la through targeting metabolic and epigenetic pathways influences CD8^+^ T cell effector functions, including enhancing antitumor immunity in preclinical models 39, 40. In addition, tumor-derived lactate promotes cancer development by modifying MOESIN lactylation, which in turn boosts TGF-beta signaling in regulatory T cells 65. In conclusion, T-cell-mediated immunity is blunted by lactylation and high lactate, facilitating tumor immune escape.

#### 2.3.3. Interaction between lactylation and immune checkpoints

Prostate cancer, particularly in its advanced and castration-resistant stages, is characterized by a highly immunosuppressive TME. Immune checkpoints, such as PD-1/PD-L1 and CTLA-4, play a pivotal role in modulating the immune response, allowing prostate cancer cells to evade immune surveillance. Emerging evidence suggests that lactylation, a novel post-translational modification derived from lactate metabolism, may regulate the expression and function of these immune checkpoints, thereby influencing tumor progression and therapeutic resistance.

##### PD-1/PD-L1 regulation by lactylation in prostate cancer

The PD-1/PD-L1 axis is a major immune checkpoint pathway that suppresses T cell activation and facilitates immune evasion in prostate cancer 66. PD-L1 is frequently overexpressed in aggressive forms of prostate cancer, and its upregulation is associated with poor prognosis and resistance to therapy 67, 68. While the link between tumor metabolism and PD-L1 expression has been explored, the role of protein lactylation in this process is still being uncovered. Recent studies indicate that histone lactylation can epigenetically regulate the transcription of immunosuppressive genes, including PD-L1. In prostate cancer cells, lactylation of histone H3 lysine 18 (H3K18la) may enhance PD-L1 gene transcription, leading to sustained immune evasion 19, 69. This suggests that metabolic reprogramming, which leads to lactate accumulation in the prostate cancer TME, not only affects immune cell function but also directly modulates PD-L1 expression through epigenetic modifications. Beyond transcriptional control, lactylation may also influence PD-L1 at the post-translational level. PD-L1 stability is tightly regulated by ubiquitination, glycosylation, and phosphorylation, which affect its degradation and localization on the tumor cell surface 70, 71. Preliminary findings from other cancer models suggest that lactylation may inhibit PD-L1 ubiquitination, thereby preventing its degradation and prolonging its immunosuppressive effects. Lactylation regulates PD-L1 expression through the lactate receptor GPR81, which reduces intracellular cAMP levels and inhibits PKA activity, leading to the activation of the transcriptional coactivator TAZ and its interaction with TEAD, ultimately enhancing PD-L1 expression 72. This mechanism enables prostate cancer cells to upregulate PD-L1 in a lactate-enriched microenvironment, thereby suppressing T-cell activity and promoting immune evasion mechanisms in prostate cancer, this could explain why PD-L1 remains highly expressed even under metabolic stress conditions. Therapeutically, these insights open new avenues for targeting PD-L1 regulation in prostate cancer. If lactylation enhances PD-L1 expression and stability, inhibiting lactylation-associated enzymes such as p300 or targeting metabolic pathways like lactate dehydrogenase (LDH) could serve as novel strategies to reduce PD-L1-mediated immune evasion. Additionally, combining lactylation inhibitors with anti-PD-1/PD-L1 checkpoint blockade could enhance immunotherapy efficacy, particularly in prostate cancer patients who exhibit resistance to current immune checkpoint inhibitors.

##### CTLA-4 and other Inhibitory receptors in prostate cancer

CTLA-4, another key immune checkpoint, functions by competing with CD28 for binding to B7 molecules (CD80/CD86) on antigen-presenting cells, thereby suppressing T cell activation.^73^ While CTLA-4 blockade has shown success in melanoma, its role in prostate cancer remains less well-defined. Lactate enhances CTLA-4 expression in tumor-infiltrating Treg cells by promoting USP39-mediated RNA splicing in a Foxp3-dependent manner, thereby sustaining Treg function in the tumor microenvironment. This mechanism facilitates immune suppression in cancer, contributing to tumor immune evasion 74. In prostate cancer, regulatory T cells (Tregs) are abundant within the TME and play a major role in immune suppression. CTLA-4 is highly expressed on Tregs, and its activity is essential for maintaining their immunosuppressive function. Histone lactylation may promote the transcription of CTLA-4, leading to increased inhibitory signaling and enhanced Treg-mediated immune suppression. This could contribute to the failure of anti-tumor immunity in prostate cancer, particularly in patients with high Treg infiltration.

Beyond CTLA-4, additional inhibitory immune receptors such as LAG-3^75^, TIM-3^76^, and VISTA, may also be regulated by lactylation in prostate cancer. LAG-3 is frequently co-expressed with PD-1 in exhausted T cells within the prostate cancer TME, contributing to immune dysfunction 77. Lactylation may enhance LAG-3 expression through epigenetic regulation, further reinforcing T cell exhaustion. TIM-3, another checkpoint receptor, has been implicated in prostate cancer progression and resistance to therapy 76. If lactylation promotes TIM-3 signaling, it could further dampen anti-tumor immunity. Given the broad impact of lactylation on multiple immune checkpoints, targeting lactylation-related pathways could offer new therapeutic strategies for prostate cancer. If lactylation promotes CTLA-4 expression and stability, combining lactylation inhibitors with CTLA-4 blockade (e.g., ipilimumab), could improve treatment outcomes. Additionally, since lactylation may regulate multiple immune checkpoints simultaneously, a multi-target approach combining anti-PD-1/PD-L1, anti-CTLA-4, and lactylation inhibitors may be necessary to overcome immune resistance in prostate cancer.

## 3. Therapeutic Potential and Future Directions

Targeting protein lactylation in prostate cancer represents an exciting frontier with the potential to bridge metabolic regulation and immune modulation. Several strategies are being explored to harness the therapeutic potential of lactylation, including metabolic interventions, enzyme inhibitors, and combination therapies.

### 3.1. Lactate modulation and inhibition restrains prostate cancer progression

Reducing lactate levels is a primary strategy to indirectly modulate protein lactylation. LDH inhibitors targeting lactate production have shown promise in preclinical models (Table 1). For example, LDH inhibitors like FX11 (LDHA inhibitor) have been demonstrated to reduce tumor growth by disrupting glycolysis-driven lactate production 78, 79. Inhibiting LDHA with GSK2837808A (GSK, an LDHA inhibitor) effectively blocks aerobic glycolysis in cancer cells, creating a high-glucose, low-lactate environment. This satisfies the glucose demands of CD8^+^ tumor infiltrating cells while destabilizing Treg cells, thereby suppressing tumor progression 80. Additionally, inhibitors of lactate production or transport, such as those targeting LDH or MCTs, may indirectly impact lactylation by reducing substrate availability 81, 82. Similarly, ketogenic diets that shift cellular metabolism away from glycolysis could potentially lower lactate availability and suppress lactylation 83, 84. These strategies decrease the metabolic support for tumor growth while simultaneously mitigating lactate-driven immune suppression.

Since lactylation is controlled by a series of enzymes, targeting these enzymes provides new strategies to modulate lactylation levels in both tumor and immune cells, enhancing the efficacy of existing therapies. For example, inhibitors targeting HATs such as p300 reduce both histone and non-histone lactylation. Small molecules like C646, a p300/CBP inhibitor, have been shown to block the activity of p300, thereby reducing lactylation^85^. Additionally, sirtuins, such as SIRT1, could be targeted with small molecules like sirtuin activators to LDHA inhibitors, such as oxamate, could allow for precise modulation of lactylation at the molecular level. *β-catenin* Docetaxel is a highly effective chemotherapy drug for treating castration-resistant prostate cancer (CRPC). Inhibition of LDHA enhances docetaxel-induced cytotoxicity, particularly in CRPC cells 86. Inhibition of LDHA by oxamate reduced H3K18la levels and circumvented immune evasion of cancer cells by enhancing CD8+ T-cell cytotoxicity 41. Furthermore, evodiamine, a natural alkaloid, has been shown to inhibit lactate-induced histone lactylation, increase Sema3A expression, and reduce angiogenesis, evodiamine could be a promising candidate for prostate cancer therapy, providing a metabolic-epigenetic approach to overcoming resistance 19.

### 3.2. Combination therapies with immunotherapy

Given the increasing evidence linking lactylation to immune evasion, combining immune checkpoint inhibitors with lactylation-targeting agents presents a promising strategy to overcome this resistance and improve treatment outcomes.

#### 3.2.1. LDHA inhibition improves the efficacy of anti-PD-1 therapy

Preclinical studies in other cancer types have demonstrated the potential of combining metabolic inhibitors with ICIs. For example, in models of melanoma and non-small cell lung cancer (NSCLC), the combination of LDH inhibitors with PD-1/PD-L1 inhibitors has shown synergistic effects, resulting in improved anti-tumor responses compared to either therapy alone. The inhibition of LDHA enhances the efficacy of anti-PD-1 treatment by boosting the anti-tumor immune response. In mouse models with LDHA-deficient melanoma tumors, there was a significant increase in the infiltration of natural killer cells and CD8^+^ cytotoxic T cells, leading to elevated IFN-γ expression. Additionally, anti-PD-1 therapy in these tumors enhanced mitochondrial activity and increased reactive oxygen species (ROS) levels. These findings demonstrate that LDHA knockdown or inhibition improves the therapeutic effectiveness of anti-PD-1 treatment 96. Reversely, cancer patients with glycolysis and lactate accumulation may benefit from PD-1/PD-L-1-based immunotherapy 32.

Moreover, recent studies have extended this concept to other treatment modalities. In particular, combining LDHA inhibition with chemotherapy and targeted therapies has shown promising synergistic effects. For example, inhibition of LDHA in combination with chemotherapy agents like cisplatin has been demonstrated to enhance the therapeutic efficacy by reprogramming the metabolic state of the tumor microenvironment, making the tumor cells more susceptible to chemotherapeutic agents 97. This synergistic effect is partly attributed to enhanced apoptosis and impaired DNA damage repair mechanisms when glycolysis is suppressed. Additionally, the combination of lactylation-targeting agents with tyrosine kinase inhibitors (TKIs) has shown potential in overcoming the resistance mechanisms often associated with TKIs. Studies have indicated that inhibiting lactate dehydrogenase A (LDHA) in conjunction with TKIs such as imatinib enhances the efficacy of treatment in chronic myeloid leukemia (CML), where lactate accumulation is known to promote drug resistance 98. This dual-target approach works by reducing the lactylation of key regulatory proteins that affect the drug efflux pumps, thereby improving the penetration and effectiveness of the TKI treatment. In conclusion, the combination of lactylation-targeting therapies with immune checkpoint inhibitors represents a promising strategy to overcome immunotherapy resistance and improve treatment outcomes in prostate cancer.

#### 3.2.2. Combination of targeting oncogene and CAR-T therapy exerts better effects

Metabolic reprogramming in tumors significantly impacts the efficacy of adoptive cell therapies such as CAR-T therapy 99. Given the crucial role of lactylation in immune evasion, targeting metabolic pathways in combination with CAR-T therapy has emerged as a promising strategy. One example is the inhibition of LDHA using oxamate, which enhances the efficacy of CAR-T therapy by suppressing CCR8 lactylation. CCR8 is predominantly expressed on Tregs within the TME, where its lactylation promotes an immunosuppressive phenotype. By blocking CCR8 lactylation, LDHA inhibition reduces Treg-mediated immunosuppression, thereby improving CAR-T cell persistence and cytotoxicity against tumors 100.

In addition, six transmembrane epithelial antigen of the prostate 1 (STEAP1) is highly expressed in in metastatic state and demonstrated as a promising therapeutic target in prostate cancer, STEAP1-directed chimeric antigen receptor (CAR) T cells demonstrate effective antitumor activity, even in low antigen density, and exhibit safety in preclinical models. Combining STEAP1 CAR-T cell therapy with tumor-localized IL-12 therapy enhances treatment efficacy by remodeling the immunosuppressive tumor microenvironment and overcoming STEAP1 antigen escape 101. STEAP1 CAR-T cell therapy is currently undergoing a clinical trial in combination with enzalutamide for the treatment of patients with metastatic castration-resistant prostate cancer (Clinical Trial ID: NCT06236139). This Phase I/II clinical trial aims to evaluate the safety and efficacy of enzalutamide in combination with STEAP1 CAR-T cell therapy.

Beyond STEAP1, other oncogenes and metabolic targets are being explored for synergistic CAR-T therapies. For example, prostate-specific membrane antigen (PSMA)-targeted CAR-T cells have demonstrated enhanced efficacy when combined with metabolic inhibitors such as dichloroacetate (DCA), which shifts tumor metabolism from glycolysis to oxidative phosphorylation, thereby reducing lactate accumulation and improving T cell functionality 102. Similarly, co-targeting fibroblast activation protein (FAP) and prostate stem cell antigen (PSCA) with dual-antigen CAR-T cells has been shown to enhance tumor clearance while reducing the risk of antigen escape 103. Overall, integrating metabolic reprogramming strategies with oncogene-targeted CAR-T therapy provides a novel avenue to enhance CAR-T cell persistence, mitigate immune suppression, and improve treatment outcomes in prostate cancer and other malignancies.

#### 3.2.3. Targeting lactate metabolism enhances the efficacy of radiotherapy

Lactate metabolism plays a crucial role in shaping the tumor microenvironment and influencing the response to radiotherapy. High lactate levels in tumors contribute to radioresistance by promoting DNA repair and reducing oxidative stress. Targeting LDHA, a key enzyme in lactate metabolism, has been shown to sensitize tumors to radiotherapy. For instance, the inhibition of LDHA using oxamate significantly enhances radiosensitivity in cancer cells, leading to increased apoptosis and autophagy following ionizing radiation (IR). Mechanistically, LDHA inhibition leads to intracellular ATP depletion, accumulation of reactive oxygen species (ROS), and impaired DNA damage repair, ultimately potentiating the effects of radiotherapy 104. Given the role of lactylation in modulating tumor immune evasion, combining lactate metabolism inhibitors with radiotherapy and immune checkpoint inhibitors (ICIs) could represent a promising strategy to improve treatment efficacy.

#### 3.2.4. Dual inhibition of lactylation and immunosuppressive pathways enhances anti-tumor immunity

Lactylation not only promotes tumor progression but also contributes to an immunosuppressive tumor microenvironment by modifying key immune regulatory proteins 105. Recent studies indicate that dual targeting of lactylation and immunosuppressive pathways may synergistically enhance anti-tumor immunity 106. For example, inhibiting LDHA or lactate transporters in combination with anti-CTLA-4 therapy leads to increased infiltration of cytotoxic T cells and reduced regulatory T cells, thereby improving anti-tumor immune responses. Furthermore, lactylation of key immune checkpoints such as PD-L1 has been implicated in immune evasion, and targeting lactylation could enhance the efficacy of PD-1/PD-L1 blockade 107. Combining metabolic reprogramming strategies that target lactylation with small molecule inhibitors of immunosuppressive pathways, such as indoleamine 2,3-dioxygenase (IDO) inhibitors, presents a promising therapeutic approach to overcoming resistance to immunotherapy in prostate cancer 8.

In conclusion, the combination of lactylation-targeting therapies with immune checkpoint inhibitors represents a promising strategy to overcome immunotherapy resistance and improve treatment outcomes in prostate cancer. However, the clinical translation of lactylation-targeting therapies is not without challenges. These therapies must be critically evaluated for their specificity and potential toxicity. Off-target effects could pose significant risks, and there is a need for further research to optimize the precision of these treatments. Current clinical trials investigating metabolic inhibitors and immunotherapies indirectly related to lactylation provide valuable insights, but many hurdles remain, such as ensuring minimal side effects and assessing long-term safety. Understanding these challenges is crucial for advancing lactylation-targeting agents from preclinical studies to clinical applications.

## 4. Conclusions and Perspective

Prostate cancer is a highly heterogeneous disease characterized by a complex cellular network and an immunosuppressive TME 108, 109. Protein lactylation represents a transformative concept in prostate cancer biology, offering a direct link between metabolic reprogramming and immune regulation. As a PTM driven by the metabolic byproduct lactate, lactylation influences a wide range of cellular processes, from gene expression and signaling to immune cell function 20, 110, 111. Its dual role as a facilitator of tumor progression and a modulator of the immune landscape positions lactylation as a critical node in prostate cancer pathogenesis 112. The integration of lactylation-targeting strategies into therapeutic regimens holds immense potential to overcome the limitations of current treatments, particularly immunotherapy 113, 114. By addressing the metabolic underpinnings of immune evasion, interventions aimed at modulating lactylation could transform prostate cancer from an immunologically “cold” tumor to a “hot” one, amenable to immune-based therapies 115. Furthermore, the identification of lactylation as a biomarker could enhance personalized treatment approaches, allowing clinicians to tailor therapies based on a patient's unique metabolic and immunological profile 116.

While significant progress has been made in understanding lactylation, much remains to be explored. Elucidating the molecular mechanisms governing lactylation in different cellular contexts (Questions 1 and 3) is essential to uncover how this modification influences distinct TME and the immune cells within them. Investigating how lactylation interacts with other PTMs, such as acetylation or phosphorylation (Question 2), will help delineate its role within broader regulatory networks and may reveal new synergistic or antagonistic pathways relevant to tumor progression and therapeutic resistance. Furthermore, understanding the tissue-specific effects of lactylation (Question 3) and its impact on immune cell function across different cancer types, including prostate cancer, is necessary for designing targeted interventions. Recent evidence suggests that lactylation may also play pivotal roles in the progression of other malignancies such as breast cancer, lung cancer, and melanoma. In breast cancer, histone lactylation driven by elevated glycolysis promotes c-Myc expression, which in turn regulates alternative splicing via SRSF10, thereby facilitating tumor progression 117. In non-small cell lung cancer, H3K18 lactylation promotes immune evasion by upregulating the POM121/MYC/PD-L1 axis, highlighting its role in tumor progression and therapeutic resistance 41. These findings indicate that lactylation is not restricted to prostate cancer but represents a broader mechanism of oncogenic regulation, further supporting its potential as a universal therapeutic target across diverse cancer types. Finally, identifying biomarkers of lactylation and assessing the systemic and off-target effects of targeting its pathways (Question 4) are critical steps toward translating these discoveries into clinical applications. These efforts will not only enhance precision medicine strategies but also address safety concerns, ensuring that therapeutic interventions are both effective and feasible. Together, these questions represent crucial directions for future research that could reshape our approach to prostate cancer treatment and beyond.

- What are the precise molecular mechanisms by which protein lactylation influences the functional states of specific immune cells, such as tumor-associated macrophages, T cells, and dendritic cells, within the prostate cancer microenvironment?
- How does lactylation interact with other PTMs, such as acetylation or phosphorylation, to regulate key processes in prostate cancer progression and therapeutic resistance?
- What are the tissue-specific effects of lactylation, particularly in modifying immune cell function across different tumor microenvironments, including prostate cancer?
- What are the potential systemic effects and off-target consequences of targeting lactylation pathways in prostate cancer, and can specific biomarkers of lactylation be identified and validated for predicting treatment responses or disease progression?

The development of robust tools to study lactylation, including high-specificity antibodies, advanced mass spectrometry techniques, and *in vivo* models of lactylation manipulation, will be instrumental in advancing this field. Furthermore, understanding the pharmacodynamics of lactylation-targeting agents, their potential toxicity profiles, and their ability to synergize with existing therapies will be key challenges to overcome before clinical translation.

In conclusion, the study of protein lactylation in prostate cancer is not merely an academic exercise but a gateway to innovative therapies that could improve outcomes for patients with this challenging malignancy. Unraveling the complexities of lactylation and translating these findings into clinical applications could mark the start of a new era in prostate cancer treatment. Here, metabolic and immunological insights converge to provide hope and precision in oncology. However, to fully realize the therapeutic potential of lactylation targeting, critical questions remain that must be addressed through continued research and collaborative efforts across scientific disciplines.

## Figures and Tables

**Table 1:** Small-molecule inhibitors and antibodies targeting lactate metabolism in cancer

Target Class	Name	Cancer	Working Mechanisms	Reference
LDHA Inhibitors	FX11	Pan-cancer	Inhibits lactate dehydrogenase A (LDHA), reducing lactylation and glycolysis-dependent tumor growth	87
	Galloflavin	Breast, prostate cancer	LDHA inhibitor that suppresses lactate production and disrupts the immunosuppressive tumor microenvironment (TME)	88
	Gossypol	Prostate, lung cancer	LDHA inhibitor that disrupts tumor metabolism and enhances T cell-mediated immune responses	89
	Stiripentol	Gastric cancer	LDHA inhibitor that blocks lactate production, inhibits lactylation of NBS1, weakens DNA repair, and overcomes tumor resistance to radiotherapy and chemotherapy	90
MCT Inhibitors	AZD3965	Lung, lymphoma	Monocarboxylate transporter 1 (MCT1) inhibitor that blocks lactate export, leading to intracellular acidification and reduced PD-L1 expression	89
	MCT4 Inhibitors	Triple-negative breast cancer	Blocks lactate export, leading to decreased extracellular lactate accumulation and improved immune activation	91
Hexokinase II Inhibitors	3-Bromopyruvate (3-BP)	Liver, pancreatic cancer	Inhibits glycolysis by targeting hexokinase II, reducing lactate production and impairing tumor immune evasion	92
HIF-1α Inhibitors	HIF-1α inhibitors	Glioblastoma, renal cancer	Suppresses lactylation-driven expression of immune checkpoint molecules such as PD-L1 and CTLA-4	93
AARS1 Inhibitors	AARS1 Inhibitors	Multiple cancers	Inhibit alanyl-tRNA synthetase (AARS1), reducing lactylation of p53 and restoring its tumor suppressor function	94
Other Targets	Monoclonal anti-LDHA antibody	Breast, ovarian cancer	Directly targets LDHA, reducing lactylation-mediated PD-L1 expression and restoring immune surveillance	95
	MCT1	Not specified	Block lactylation of cGAS, restoring its function in antitumor immunity	94

## Abbreviations

CAF: cancer-associated fibroblasts
CRPC: castration-resistant prostate cancer
DCA: dichloroacetate
DFS: disease-free survival
HATs: Histone acetyltransferases
HIF-1α: Hypoxia-inducible factor 1-alpha
ICIs: immune checkpoint inhibitors
IR: ionizing radiation
KAT2A: Lysine acetyltransferase 2A
KAT5: Lysine acetyltransferase 5
Kce: N-ε-carboxyethyl lysine
KD-la: D-lysine lactylation
KL-la: L-lysine lactylation
LDHs: lactate dehydrogenases
LDs: lipid droplets
LRGs: Lactylation-related genes
MCTs: monocarboxylate transporters
NK: natural killer
NSCLC: non-small cell lung cancer
PTM: post-translational modification
ROS: reactive oxygen species
STEAP1: six transmembrane epithelial antigen of the prostate 1
TAMs: tumor-associated macrophages
TCA: tricarboxylic acid
TME: tumor microenvironment
